# Using high pressure to understand the behavior of organic molecular crystals

**DOI:** 10.1107/S2052252524012478

**Published:** 2025-01-01

**Authors:** María Guadalupe Vasquez-Ríos

**Affiliations:** a Département de Chimie Université de Sherbrooke Sherbrooke Québec Canada

**Keywords:** organic molecular crystals, high pressure, polymorphism, polycyclic aromatic hydrocarbons, benzo[*a*]pyrene, density functional theory

## Abstract

In the recent publication by Zhou *et al.* [(2025). *IUCrJ*, **12**, 16–22], the crystal structure of benzo[*a*]pyrene was studied under high pressure up to 28 GPa using single-crystal X-ray diffraction and DFT calculations. This commentary highlights the importance of high pressure for analyzing organic molecular crystals.

In this issue of **IUCrJ**, Zhou and coworkers (Zhou *et al.*, 2025 ▸) present a study on the behavior of the crystal structure of benzo[*a*]pyrene (B*a*P) under high pressure (up to 28 GPa) using *in situ* synchrotron single-crystal X-ray diffraction (SCXRD) in diamond anvil cells (DACs) with a He pressure medium. Density functional theory (DFT) calculations were conducted to support the experimental results. The article by Zhou *et al.* (2025 ▸) is one of very few reports analyzing organic molecular crystals above 10 GPa. Importantly, this methodology could be applied more broadly to other crystal structures of organic compounds.

Organic molecular crystals display a range of non-covalent interactions (*e.g.* hydrogen bonds, π−π stacking, van der Waals contacts). Applying pressure to crystalline organic solids provides insights into understanding the nature of these interactions (Hemley & Dera, 2000 ▸). In the last few years, one area that has dominated the field of high-pressure solid-state chemistry is the search for new polymorphs, in which the challenge relies on understanding the factors that control the formation of crystal structures, their stability, and transformations (Boldyreva, 2007 ▸). Polymorphic forms provide an opportunity to investigate structure–property relationships (Bernstein, 1993 ▸). However, in contrast to inorganic compounds, high-pressure SCXRD studies on organic molecular crystals are primarily limited by complexities involving X-ray diffraction techniques. Notable advances in synchrotron SCXRD in DACs has demonstrated that an inert gas pressure medium, such as He or Ne, extends the scope for analyzing this class of organic materials (Dubrovinsky, 2013 ▸). So far, analyses of crystalline organic solids above 10 GPa are scarce. Some exceptional examples involve the study of the crystal structures of anthracene (up to 27.8 GPa; Oehzelt *et al.*, 2003 ▸), l-threonine (up to 22 GPa; Giordano *et al.*, 2019 ▸), chrysene (up to 20 GPa; Zhao *et al.*, 2024 ▸) and pyrene (up to 35 GPa; Zhou *et al.*, 2024 ▸). Nevertheless, most of these transformations are liquid-assisted, *e.g.* crystallization of pure liquids at high pressures, crystallization at high pressures from solutions *etc*. (Boldyreva, 2007 ▸). The direct compression of the crystalline material is less frequent, but this method has been applied in the study of small molecules (*i.e.*, considering the conformational flexibility). Despite significant efforts, the analysis of large organic molecules remains a challenge.

In the work of Zhou *et al.* (2025 ▸), the authors undertake these challenges and identify two previously unknown polymorphs of B*a*P on compression up to 28 GPa ▸. Having five benzene rings, B*a*P is one of the most studied of the polycyclic aromatic hydrocarbons (PAHs) owing to its high toxicity [being carcinogenic, teratogenic and mutagenic (Fu *et al.*, 2022 ▸)]. PAHs are widespread throughout the universe (Li, 2020 ▸); they are ubiquitous organic molecules formed by the incomplete combustion of matter. B*a*P and other PAHs are of concern due to their multiple effects on human health. Over recent years, great efforts have been devoted to studying this class of organic compounds under extreme thermodynamic conditions (Oehzelt *et al.*, 2003 ▸; Fabbiani *et al.*, 2006 ▸; Zhao *et al.*, 2024 ▸; Zhou *et al.*, 2024 ▸). Detailed studies of their crystal structures are essential for understanding the molecular basis of their chemical properties. Moreover, such information will provide insight into the evolution of biological processes that occur on an astronomical scale.

In the article by Zhou *et al.* in this issue, B*a*P-I single crystals were put into DACs at room temperature and then compressed at ambient pressure and 2.2 GPa. Further analysis of the B*a*P-I crystal structure confirmed the monoclinic space group *P*2_1_/*c*, as reported previously (Carrell *et al.*, 1997 ▸). Upon compression at 4.8 GPa, a phase transition from B*a*P-I to B*a*P-II was observed. Although the intermolecular angle differed, the structure remained with the same space-group symmetry. Then a second phase transition from B*a*P-II to B*a*P-III occured at 7.1 GPa, accompanied by a change to the triclinic space group *P*1. Thus, in the transition from phase I to phase III the molecular arrangement decreases in crystallographic symmetry. Further information on the experimental procedure is provided in the authors’ supporting information.

To provide an understanding of the structural changes, the authors performed DFT calculations to demonstrate the compressional behavior of B*a*P polymorphs and how the compression affects the energy gap between them. The analysis of the calculated and experimental data for the unit-cell volumes for B*a*P polymorphs up to ∼28 GPa suggests a transition accompanied by a modification of the unit-cell parameters associated with a change of space-group symmetry. Strikingly, the enthalpy differences (Δ*H*) revealed that B*a*P-III is the most stable polymorph beyond 3.5 GPa. A geometrical analysis was also carried out, in terms of intermolecular distances (*d*) and interplanar angles (δ). The phase transition from B*a*P-I to B*a*P-III displays a decrease of the intermolecular distances, thus resulting in a more efficient packing of the molecules. The intermolecular angles also changed; the B*a*P molecules became flatter on compression.

Whereas the effect of pressure on hydrogen bonds in organic molecular crystals has been discussed in numerous publications (Boldyreva, 2003 ▸), analysis involving conjugated organic molecules such as PAHs has been addressed less (Fabbiani *et al.*, 2006 ▸; Zhou *et al.*, 2024 ▸). In the article by Zhou *et al.* in this issue, the authors have further examined the evolution of intermolecular interactions in the B*a*P structure on compression using Hirshfeld and shape index surfaces (McKinnon *et al.*, 2004 ▸). The results indicate that the contributions of C⋯C interactions in π–π stacks increase, while the contributions of H⋯H interactions for the van der Waals contacts decrease. Indeed, the results would suggest that pressure enhances the contributions of π–π contacts.

In summary, the article by Zhou and coworkers demonstrates the impact of high pressure above 10 GPa using *in situ* synchrotron SCXRD in DACs with a ‘soft medium’ such as He for the analysis of the crystal structure of B*a*P. Combined with computational methods, an effective methodology is described for predicting polymorphs under high pressure. This approach may be useful for analyzing organic molecular crystals of pharmaceutical interest (*i.e.*, for the discovery and development of new drugs) and further applications in materials science.

## Figures and Tables

**Figure 1:**
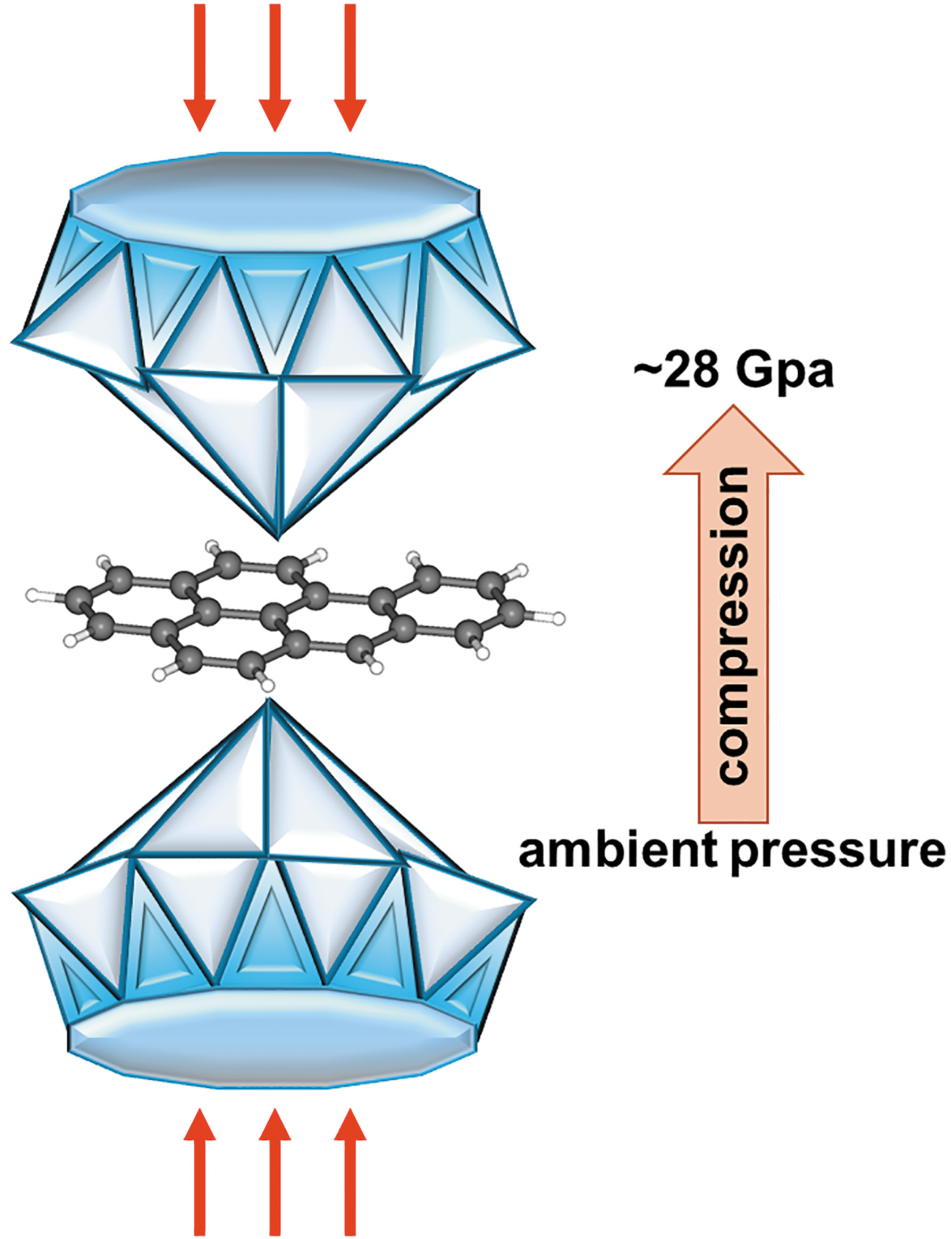
High-pressure behavior of B*a*P between ambient pressure and ∼28 GPa.
